# Racial differences in testicular cancer in the United States: descriptive epidemiology

**DOI:** 10.1186/s12885-020-06789-2

**Published:** 2020-04-06

**Authors:** Yang Li, Qi Lu, Yu Wang, Shuangge Ma

**Affiliations:** 1 Center for Applied Statistics, Renmin University of China, Beijing, China; 2 School of Statistics, Renmin University of China, Beijing, China; 3 School of Public Health, Yale University, New Haven, Connecticut USA

**Keywords:** Testicular cancer, Racial differences, SEER

## Abstract

**Background:**

Testicular cancer (TC) is the most common malignancy in young adult men, and in many countries the incidence rates of testicular cancer have been increasing since the middle of the twentieth century. Since disease presentation and tumor progression patterns are often heterogeneous across racial groups, there may be important racial differences in recent TC trends.

**Methods:**

In this study, Surveillance, Epidemiology, and End Results (SEER) data on TC patients diagnosed between 1973 and 2015 were analyzed, including the following racial/ethnic groups: non-Hispanic whites (NHW), Hispanic whites (HW), blacks, and Asians and Pacific Islanders (API). Patient characteristics, age-adjusted incidence rates, and survival were compared across racial groups. A multivariate Cox model was used to analyze the survival data of TC patients, in order to evaluate racial differences across several relevant factors, including marital status, age group, histologic type, treatment, stage, and tumor location.

**Results:**

NHWs had the highest incidence rates, followed by blacks, HWs, and APIs. There were significant survival differences among the racial groups, with NHWs having the highest survival rates and blacks having the lowest.

**Conclusion:**

An analysis of SEER data showed that racial differences existed among TC patients in the United States with respect to patient characteristics, incidence, and survival. The results can be useful to stakeholders interested in reducing the burden of TC morbidity and mortality.

## Background

Testicular cancer (TC) is the most common malignancy in young men, and an increase in the incidence of TC has been reported in recent years [1–3]. The age-standardized incidence of TC has been reported to vary across European countries, and based on registry data, has increased annually at rates ranging from 2.3% (in Sweden) to 5.2% (in East Germany) [4]. Other studies have shown that the age-standardized incidence of TC is greatest in western Europe (7.8%), northern Europe (6.7%), Australia (6.5%), and North America (5.1%), while the lowest incidences were found in Asia (0.5–1.5%) and Africa (0.2–0.7%) [5, 6]. Established risk factors for TC include a family history of the disease, previous germ cell tumor, subfertility, undescended testis, testicular microlithiasis and the presence of small foci of intratesticular calcification [7, 8]. The peak age of TC incidence is 30 years old, and evidence suggests that in utero and early life exposures may be important contributors to TC etiology. Numerous postnatal factors have also been reported to influence TC risk, including injury, infection, occupational factors, and hormonal exposures, especially those experienced during periadolescence [9, 10].

Three histological types, germ cell tumors (GCTs), teratoma and embryonal carcinoma (EC), were analyzed in this paper. GCTs are the most common histological type of TC, comprising more than 95% of all TC cases [11–13]. GCT can be further divided into two subtypes: seminomas and non-seminomas, with the latter found more frequently at younger ages, occurring predominantly among adolescent men (age 15–19 years) [14]. Apart from GCTs, two additional histological types were included in this analysis, teratoma and embryonal carcinoma (EC), which each have peak incidence rates among different age groups. Within the teratoma group there are two periods of peak incidence, one occurring among young children, age 1–2 years old, and the other occurring among young adults, around age 25–35 years. Most patients with teratoma present with a painless mass that is hard, nodular, or irregular [15]. ECs originate from primordial germ cells with multiple differentiation potential, leading to highly malignant tumors with a peak incidence in childhood, and a second peak occurring among adults age 30–40 years [16]. Teratoma and EC are rare neoplasm affecting the pediatric population and they have classically been reported to be the second most common testis tumor in children [17]. Therefore, the study of teratoma and EC can analyze the current situation of testicular cancer in American children, and provide reference for the diagnosis and early detection of testicular cancer in children.

The prognosis following a TC diagnosis depends on stage and therapeutic approach. Post-orchidectomy options include active surveillance, radiotherapy or single-agent chemotherapy. About 15% of men with stage I disease will relapse within 4 years [18]. Salvage rates are high, so active surveillance has the advantage of avoiding unnecessary treatment and associated adverse effects. Radiotherapy is administered to the ipsilateral renal hilum, pelvic lymph nodes, and the bilateral para-aortic nodes, as well as the regional lymph nodes of the involved testis. Five-year survival rates have increased significantly over the last 30 years from about 63% to more than 90%. This change is attributable to improved therapy for patients with disseminated TC other than seminoma [19].

Some research paid attention to the ethnic differences in testicular cancer. Previous studies have shown that the incidence of testis cancer varies substantially with ethnicity, and is two to five-fold higher among US non-Hispanic whites than among Hispanic whites, Asians, and African-Americans [3, 20]. But previous research on racial differences among TC patients is insufficient, particularly since many available TC studies are limited to analyses focused on specific racial groups (e.g. Hispanic and non-Hispanic white patients) [21], or specific outcomes (e.g. incidence only [22]). The goal of this study was to provide a comprehensive description of racial differences among TC patients in the United States using data from the SEER registry system, and approached from different angles, including analyses of patients’ characteristics, clinic pathologic features, incidence, and survival rates. The current analysis includes both a wider spectrum of racial groups, including non-Hispanic whites (NHWs), Hispanic whites (HWs), blacks, and Asians and Pacific Islanders (APIs), as well as a multifactorial approach, including data on patient characteristics, clinic pathological features, incidence rates, and survival rates. This study will provide more information for the diagnosis, prevention and treatment of TC and will be useful to TC epidemiologists, clinicians and policy makers.

## Methods

### Source population

The population-based sample was obtained from the Surveillance, Epidemiology, and End Results (SEER) database located at http://seer.cancer.gov/. The SEER 9, 13, and 18 registries were analyzed in this study, which cover approximately 9.5, 14, and 28% of the population in the United States, respectively. TC cases were identified by the International Classification of Diseases for Oncology (ICD-O-3) site codes C620-C629. Tumor sites and histology types were coded according to criteria specified by the WHO in ICD-O-3. The ICD-O-3 site codes C620, C621, and C629 represent the tumor sites undescended testis, descended testis, and NOS, respectively. Histologic types are also grouped using the ICD-O-3 code, with patients with GCT, teratoma, and EC identified by the histology codes 9060–9065, 9080–9085, and 9070–9072, respectively. There were three main treatment modalities: surgery, radiotherapy, and chemotherapy, that were each analyzed alone or in combination, along with a separate category for active surveillance (no initial treatment).

For the analysis of patient characteristics and clinic pathological features, we used SEER 9, which contains data on cancers diagnosed between 1973 and 2015, as well as information on marital status, age at diagnosis, age group (0–39 years, 40–64 years, 65+ years), survival time, stage (in situ, localized, regional, and distant), and lymph node involvement (nodal, extra nodal). For the analysis of incidence rates, we used SEER 9 data, containing detailed race and incidence information for cancers diagnosed between 1973 and 2015. For the analysis of survival rates, we used SEER 9 data, which contains information on cancers diagnosed between 1973 and 2010, with follow-up until December 31, 2015.

### Statistical analysis

Chi-squared tests and ANOVA were used to compute *P*-values for the comparison of patient characteristics and clinic pathologic features across racial groups. The analysis was conducted using SAS version 9.4. We also used SEER*Stat software and United States 2000 Census data to calculate age-adjusted incidence rates and five-year relative survival rates. When adjusting for marital status, age group, stage, location, and treatment strategy, we conducted multivariate Cox regression analyses.

We used multivariate Cox model to analyze the survival data of TC patients. The specific form of the model is as follows:

$$ h\left(t,X\right)={h}_0(t)\exp \left({\beta}_1{X}_1+{\beta}_2{X}_2+{\beta}_3{X}_3+{\beta}_4{X}_4+{\beta}_5{X}_5\right) $$

*X*_1_: Marital status; *X*_2_: Age group; *X*_3_: Stage; *X*_4_: Location; *X*_5_: Treatment strategy;

*β*: Partial regression coefficients of independent variables.

## Results

### Patient characteristics and clinic pathologic features

The main findings related to testicular cancer patient characteristics and clinic pathologic features, for both the full cohort and among the various racial groups, are outlined in Table 1. Most TC patients were married (47.5%), but there were significant differences in marital status distribution across racial groups (*p* < 0.001). Age at diagnosis was also significantly different across races (*p* < 0.001). NHWs had the oldest age at diagnosis (37.0 years) while HWs had the youngest age at diagnosis (30.8 years). For all patients combined, the median survival time after diagnosis was 109.0 months. NHWs had the longest survival time, while HWs had the shortest survival time. Furthermore, there were statistically significant racial differences in subtype distribution (*p* < 0.001). For all patients, seminomas were the most common subtype (52.4%). Seminoma and EC proportions were highest among NHWs (54.1 and 11.9%, respectively), whereas the proportion of teratomas and non-seminomas were highest among HWs (32.5 and 3.9%, respectively).

**Table 1:** Testicular cancer patient characteristics and clinicopathologic features for the full cohort and among different racial groups

	Total (*n* = 56,898)	NHW (*n* = 42,556)	HW (*n* = 9282)	Black (*n* = 1602)	API (*n* = 2607)	*P*-value
**Marital status**						< 0.001
Single	23,707(42.2)	16,341(38.4)	5121(55.2)	877(54.7)	1298(49.8)	
Married	26,663(47.5)	21,624(50.8)	3383(36.5)	516(32.2)	1080(41.4)	
Separated/D/W	3518(6.3)	2850(6.7)	425(4.6)	118(7.4)	118(4.5)	
**Age group**						< 0.001
0–39	38,408(68.4)	27,663(65.0)	7731(83.3)	1051(65.6)	1846(70.8)	
40–64	15,309(27.2)	12,293(30.1)	1374(14.8)	483(30.1)	609(23.4)	
65+	2475(4.4)	1766(4.8)	177(1.9)	68(4.2)	152(5.8)	
**Age at diagnosis**	35.9 ± 17.2	37.0 ± 15.1	30.7 ± 15.3	36.2 ± 14.2	35.1 ± 14.8	< 0.001
**Survival time**	109.0	124.0	67.0	84.5	85.0	< 0.001
**(months)**	(41.0,191.0)	(51.0,214.0)	(21.0,135.0)	(27.5161.0)	(29.0,174.0)	
**Subtype**						< 0.001
Seminomas	29,423(52.4)	23,018(54.1)	4165(44.9)	835(52.1)	1321(50.7)	
Teratoma	13,947(24.8)	9945(23.4)	3013(32.5)	320(20.0)	635(24.4)	
ECs	6447(11.5)	5079(11.9)	977(10.5)	121(7.6)	256(9.8)	
Non-seminomas	1521(2.7)	1010(2.4)	366(3.9)	60(3.8)	81(3.1)	
Others	4854(8.6)	3504(8.)	761(8.2)	266(16.6)	314(12.0)	
**Location**						< 0.001
Undescended testis	969(1.7)	611(1.4)	208(2.2)	61(3.8)	88(3.4)	
Descended testis	19,451(34.6)	13,699(32.2)	4378(47.2)	448(28.0)	891(34.2)	
Testis (NOS)	35,772(63.7)	28,246(66.4)	4696(50.6)	1093(68.2)	1628(62.5)	
**Stage**						< 0.001
In situ	97(0.2)	63(0.2)	22(0.2)	9(0.5)	3(0.1)	
Localized	36,513(65.0)	28,136(66.2)	5718(61.6)	919(57.4)	1638(62.8)	
Regional	10,173(18.1)	7671(18.0)	1751(18.9)	315(19.7)	415(15.9)	
Distant	6472(11.5)	4388(10.3)	1486(16.0)	244(15.3)	340(13.0)	
**Tumor size (mm)**	45.7 ± 36.3	42.3 ± 33.2	52.4 ± 41.2	57.4 ± 44.0	53.0 ± 42.5	< 0.001
**Regional nodes**						< 0.001
All nodes negative	7693(14.4)	5948(14.6)	1124(13.1)	253(16.8)	360(14.8)	
***≥***1 nodes positive	45,676(85.6)	34,797(85.4)	7436(86.9)	1253(83.2)	2072(85.2)	
**Treatment**						< 0.001
Surgery	22,848(40.2)	16,710(39.3)	3914(42.2)	638(39.8)	1021(39.2)	
CS	15,542(27.3)	11,152(26.2)	3073(33.1)	452(28.2)	741(28.4)	
RS	15,176(26.7)	12,382(29.1)	1693(18.2)	352(22.0)	637(24.4)	
CRS	1336(2.4)	970(2.3)	214(2.3)	54(3.4)	94(3.6)	
Chemotherapy	964(1.7)	615(1.5)	236(2.5)	53(3.3)	56(2.2)	
No treatment	790(1.4)	552(1.3)	114(1.2)	42(2.6)	44(1.7)	
Radiation	119(0.2)	97(0.2)	11(0.1)	3(0.2)	4(0.2)	
CR	123(0.2)	78(0.2)	27(0.3)	8(0.5)	10(0.4)	

Cancers diagnosed between 1973 and 2015 in the SEER 9 database. Data are median (interquartile range) for survival time, mean ± standard deviation for age at diagnosis and tumor size, and count (percentage) for all categorical variables.

*NHW* non-Hispanic whites, *HW* Hispanic whites, *API* Asians and Pacific Islanders, *ECs* embryonal carcinomas, *CS* chemotherapy plus surgery, *RS* radiotherapy plus surgery, *CRS* chemotherapy plus radiotherapy plus surgery, *CR* chemotherapy plus radiotherapy

Tumor location was also significantly heterogenous across racial groups (*p* < 0.001). The most commonly provided description of tumor location was “not otherwise specified” (NOS). Blacks had the highest rates of TC NOS, and the highest proportion of tumors found in undescended testis (68.2 and 3.8%, respectively). HWs had the highest proportion of tumors in descended testis (47.2%). Localized tumors accounted for the largest proportion of the three tumor stages (65.0%). When comparing racial groups, NHWs had the highest proportion of localized tumors (66.2%), blacks had the highest proportion of regional tumors (19.7%), and HWs had the highest proportion of distant tumors (16.0%). For all patients combined, the average tumor size was 45.3 mm, but this also varied across racial groups. Blacks had the largest mean tumor size (57.3 mm) while NHWs had the smallest tumor average size (42.1 mm). Finally, there were significant racial differences in node negative distribution and treatment strategy. Blacks had the highest proportion of node-negative tumors (16.8%), while HWs had the highest proportion of TC with more than one positive lymph node (86.9%). For all patients, surgery accounted for the largest proportion of all treatments (40.2%). HWs had the highest proportion of surgery (S) and chemotherapy plus surgery (CS; 2.2 and 33.1%, respectively), NHWs had the highest proportion of radiotherapy plus surgery (RS; 29.1%), blacks had the highest proportion of chemotherapy (C), no treatment, and chemotherapy plus radiotherapy (CR; 3.3, 2.6, and 0.5%, respectively), and APIs had the highest proportion of chemotherapy plus radiotherapy plus surgery (CRS; 3.6%).

### Incidence rates

In our analysis, the age-adjusted incidence rate for all races was 2.60 cases per 100,000 person-years (Table 2), which decreased with older age. The incidence rates corresponding to the age groups 0–39 years, 40–64 years, and 65+ years were 3.09, 2.54, and 0.50 cases per 100,000 person-years, respectively. NHWs had the highest incidence rate (3.05 cases per 100,000 person-years) which, by comparison, was much higher than that of APIs and blacks (1.22 and 0.58 cases per 100,000 person-years, respectively). In analyses of the major TC subtypes, seminomas had the highest incidence rates (1.55 cases per 100,000 person-years), followed by teratomas, ECs, and non-seminomas. For all four histologic types, NHWs had the highest incidence rates (2.08, 0.94, 0.38, and 0.10 cases per 100,000 person-years, respectively), followed by HWs. Blacks had the lowest incidence rates for each of the four histologic subtypes. Figure S1 shows the trend of incidence rate from 1973 to 2012 for each racial/ethnic group. The incidence of each racial groups showed an increasing trend and non-Hispanic whites had higher growth rate.

**Table 2:** Age-adjusted testicular cancer incidence rates per 100,000 person-years for the full cohort and different racial groups, stratified by age and subtype

	Total	NHW	HW	Black	API
**All ages**	2.60(2.48–2.70)	3.05(2.87–3.19)	2.28(2.11–2.40)	0.58(0.54–0.68)	1.22(1.13–1.32)
0–39	3.09(3.04–3.14)	3.67(4.30–4.45)	2.82(2.72–2.90)	0.62(0.54–0.70)	1.49(1.28–1.56)
40–64	2.54(2.48–2.60)	2.94(2.59–3.45)	1.59(1.47–1.68)	0.68(0.58–0.78)	1.10(0.95–1.19)
65+	0.50(0.48–0.53)	0.54(0.52–0.59)	0.33(0.21–0.44)	0.17(0.11–0.24)	0.30(0.17–0.39)
**Subtype**					
Seminomas	1.55(1.52–1.57)	2.08(2.04–2.12)	1.19(1.14–1.25)	0.36(0.33–0.40)	0.60(0.56–0.65)
Teratomas	0.72(0.70–0.73)	0.94(0.91–0.96)	0.72(0.68–0.75)	0.14(0.12–0.16)	0.26(0.23–0.29)
ECs	0.27(0.26–0.28)	0.38(0.37–0.40)	0.22(0.20–0.24)	0.04(0.03–0.06)	0.10 (0.08–0.12)
Non-Seminomas	0.08(0.07–0.09)	0.10(0.09–0.10)	0.09(0.08–0.10)	0.03(0.02–0.04)	0.03(0.02–0.05)

Diagnoses occurred in the period from 1973 to 2015 using data from the SEER 9 database. Data are estimated rates (95% confidence intervals), age-adjusted using the U.S. 2000 Census population.

*NHW* non-Hispanic whites, *HW* Hispanic whites, *API* Asians and Pacific Islanders, *ECs* embryonal carcinomas

### Survival rates

**Fig. 1:**
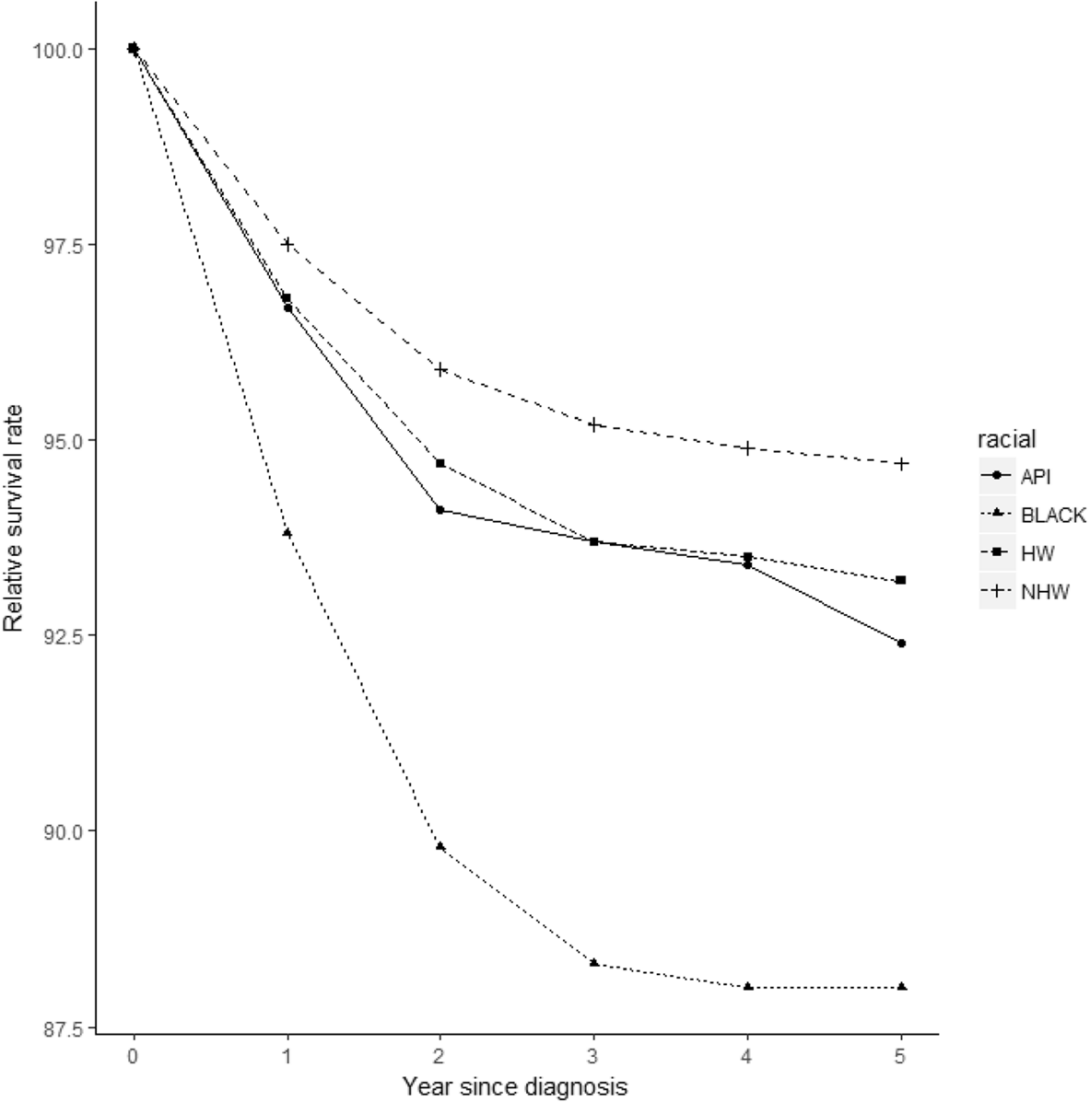
Five-year survival from the date of diagnosis, stratified by racial group. Cancers were diagnosed in the period from 1973 to 2010 with follow-up until 12/31/2015. (API: Asians and Pacific Islanders. HW: Hispanic whites. NHW: non-Hispanic whites)

Figure 1 shows the unadjusted overall survival rates for the five years after diagnosis in each racial/ethnic group. NHWs had the most favorable overall survival in all years, while blacks had much lower survival rates than other racial groups. The survival curves of the other two racial groups cross. Figure S2 shows the trend of 5-year relative survival rate from 1973 to 2007 with follow-up until 12/31/2012 for each ethnic group. The figure shows that the relative survival rates have been significantly improved for each ethnic group. The survival rate of NHWs has always fluctuated in a high range, while blacks had much lower survival rates than other racial groups.

Table 3 shows the five-year relative survival rates stratified by age group, histologic type, stage, location, and treatment. Within each age group, NHWs had the best survival and blacks had the poorest survival, and these differences were statistically significant in multivariate analyses. When stratified by stage, NHWs had the best five-year survival among patients with localized disease (98.9%), followed by HWs (98.2%), APIs (97.8%) and blacks (97.1%). For regional stage, NHWs had the best five-year survival rates (95.3%), followed by HWs (94.5%), APIs (91.0%), and blacks (87.7%). For distant stage, NHWs again had the best five-year survival rates (71.0%), followed by HWs (67.5%), APIs (62.8%), and blacks (58.4%). For all three tumor stages, the racial differences were significant according to multivariate Cox regression. When grouped by tumor location, for undescended testis, APIs had the highest survival rates (95.5%), followed by NHWs (93.7%), HWs (92.0%), and blacks (90.7%). For descended testis and testis NOS, NHWs had the best survival rates (96.8 and 94.3%, respectively). For all locations, the racial differences were significant according to multivariate Cox regression. For each of the four histologic subtypes included in the analyses, five-year survival rates were all highest in NHWs and lowest in blacks. The multivariate Cox regression analysis also indicated significant differences among histologic types. When stratified by treatment, the five-year survival rates were 89.9, 44.9, and 65.6%, for CS, CR, and CRS, respectively; and all were highest among NHWs. For chemotherapy and RS, HWs had the best survival rates (68.7 and 99.5%, respectively). For chemotherapy and radiotherapy alone, blacks had the lowest survival rates (38.2, and 50.3%, respectively). Finally, for RS and CS, APIs had the worst survival rates (98.0, and 83.8%, respectively). There were no significant differences in survival for patients receiving either surgery alone or CRS, according to the multivariate Cox model. However, for all other treatment strategies, including chemotherapy alone, radiation alone, CS, RS, and CR, racial differences were statistically significant.

**Table 3:** Five-year survival rates among testicular cancer patients from different racial groups, stratified by age, marital status, stage at diagnosis, location, and treatment

Survival rates	Total	NHW	HW	Black	API	*P*-value
**All ages**	94.4	95.0	92.3	88.8	91.7	
	(94.1–94.6)	(94.7–95.3)	(91.5–93.0)	(86.6–90.6)	(89.9–93.1)	
0–39	94.4	95.2	92.1.	88.9	92.2	0.003
	(94.1–94.7)	(94.9–95.5)	(91.2–92.8)	(86.3–91.1)	(90.3–93.7)	
40–64	94.9	95.3	93.8	88.9	92.3	0.012
	(94.4–95.4)	(94.7–95.8)	(91.6–95.4)	(84.2–92.3)	(88.3–94.9)	
65+	82.8	84.1	79.0	46.9	65.6	0.008
	(77.4–87.1)	(78.2–88.5)	(57.5–90.5)	(12.7–75.7)	(41.9–81.6)	
**Marital status**						
Single	92.1	92.9	90.3	86.2	90.0	< 0.001
	(91.7–92.5)	(92.4–93.4)	(89.2–91.4)	(83.0–88.8)	(87.4–92.0)	
Married	96.7	97.0	95.6	92.4	93.5	< 0.001
	(96.4–97.0)	(96.7–97.4)	(94.5–96.5)	(88.7–94.9)	(90.9–95.4)	
Separated/D/W	90.3	90.8	865	87.8	91.8	0.221
	(88.8–91.6)	(89.2–92.2)	(81.3–90.3)	(76.8–93.8)	(80.3–96.7)	
**Stage at diagnosis**						
Localized	98.7	98.9	98.2	97.1	97.8	0.002
	(98.5–98.9)	(98.6–99.1)	(97.5–98.7)	(94.9–98.3)	(96.2–98.7)	
Regional	94.9	95.3	94.5	87.7	91.0	0.009
	(94.3–95.4)	(94.6–95.9)	(92.8–95.8)	(82.0–91.7)	(86.3–94.1)	
Distant	69.6	71.0	67.5	58.4	62.8	< 0.001
	(68.2–70.9)	(69.4–72.5)	(64.3–70.4)	(50.6–65.5)	(55.2–69.5)	
**Location**						
Undescended	93.4	93.7	92.0	90.7	95.5	< 0.001
testis	(91.1–95.2)	(90.7–95.8)	(85.2–95.8)	(75.9–96.6)	(83.8–98.8)	
Descended	95.6	96.8	92.6	91.1	93.1	< 0.001
testis	(95.2–96.0)	(96.3–97.2)	(91.4–93.6)	(86.5–94.2)	(89.8–95.3)	
Testis (NOS)	93.8	94.3	92.1	87.8	90.7	< 0.001
	(93.5–94.1)	(94.0–94.7)	(91.0–93.1)	(84.9–90.1)	(88.5–92.5)	
**Histologic subtype**						
Seminomas	97.9	98.1	97.3	96.4	96.6	< 0.001
	(97.6–98.2)	(97.8–98.4)	(96.5–98.0)	(93.9–97.9)	(94.7–97.8)	
Teratomas	93.0	93.8	90.8	84.7	90.7	< 0.001
	(92.5–93.5)	(93.2–94.4)	(89.3–92.1)	(78.8–89.0)	(86.7–93.5)	
ECs	91.2	91.4	90.7	79.2	89.4	< 0.001
	(90.4–92.0)	(90.5–92.3)	(88.2–92.8)	(69.0–86.4)	(83.3–93.4)	
Non-seminomas	83.8	87.1	74.5	76.1	75.9	< 0.001
	(81.2–86.0)	(84.1–89.5)	(67.8–80.0)	(58.5–87.0)	(58.4–86.9)	
**Treatment**						
Surgery	97.0	97.2	96.6	91.6	96.7	0.259
	(96.5–97.4)	(96.6–97.6)	(94.6–97.9)	(86.2–95.0)	(90.2–98.4)	
Chemotherapy	57.2	56.2	68.7	38.2	58.7	< 0.001
	(51.8–62.3)	(49.8–62.0)	(53.1–80.0)	(17.4–58.9)	(80.8–89.5)	
Radiation	83.9	86.9	66.7	50.3	97.3	< 0.001
	(72.5–90.9)	(75.0–93.3)	(5.4–94.5)	(0.6–981.2)	(93.5–98.9)	
RS	99.1	99.1	99.5	98.3	98.0	0.007
	(98.7–99.4)	(98.6–99.4)	(97.5–99.9)	(93.3–99.6)	(95.0–99.2)	
CS	89.5	89.9	89.6	84.4	83.8	0.003
	(88.7–90.3)	(89.0–90.7)	(86.6–92.0)	(77.7–89.3)	(78.4–88.0)	
CR	36.0	44.9	18.8	40.2	40.7	< 0.001
	(22.0–50.1)	(26.8–61.4)	(1.1–53.7)	(5.2–75.5)	(19.8–60.8)	
CRS	63.4	65.6	59.5	65.1	43.9	0.946
	(58.4–67.9)	(60.0–70.6)	(43.2–72.6)	(37.8–82.8)	(19.9–65.7)	

Cancers were diagnosed in the period from 1973 to 2010 and followed up to 12/31/2015 using data in the SEER 9 database. Data are estimated rates (95% confidence intervals). ECs: embryonal carcinomas.

*NHW* non-Hispanic whites, *HW* Hispanic whites, *API* Asians and Pacific Islanders, *ECs* embryonal carcinomas, *RS* radiotherapy plus surgery, *CS* chemotherapy plus surgery, *CR* chemotherapy plus radiotherapy, *CRS* chemotherapy plus radiotherapy plus surgery

## Discussion

This study aimed to provide a comprehensive description of racial differences among TC patients in the United States. Previous studies described racial differences among other cancer patients, such as melanoma [23], bladder cancer [24] and so on, but no research focus on racial differences among TC patients. According to our analysis, there were significant racial differences in patient characteristics, clinic pathologic features, incidence and survival. These results may be of use to cancer epidemiologists, clinicians, and individuals and institutions interested in developing effective policies for TC treatment and prevention.

When compared to previous literature, our findings are similar to earlier reports regarding age at diagnosis, tumor grade distribution, and TC incidence rates [2, 5, 25–28]. Furthermore, our statistical analyses suggest the existence of racial differences in the distributions of gender, marital status, age at diagnosis, survival time, histologic type, stage, treatment, tumor size, nodal involvement, and tumor location among TC patients. These observed differences may reflect differences in the timing of malignant tumor diagnoses, and the importance of early detection.

A few previous publications have also studied racial differences in TC incidence [20, 29, 30]. Chia et al analyzed data from the Cancer Incidence in Five Continents study, including age-standardized incidence rates over successive 5-year time periods with data from populations in the Americas, Asia, Europe, and Oceania [31]. They found that testicular cancer incidence remained highest in northern European populations (8.0–9.0 cases per 100,000) and lowest in Asian and African populations (< 1 case per 100,000). Our analysis includes a large, racially diverse population, and therefore has the potential to generate more comprehensive results. NHWs had the highest incidence rates of TC among the four major racial groups, while blacks had the lowest. Previous studies have reported on the association between some risk factors, including cryptorchidism, history of testicular cancer, and family history of testicular cancer, and the incidence of testicular cancer [32, 33]. However, it remains unclear why the incidence of TC among APIs and blacks is so much lower than that observed among whites. Possible explanations include differences in genetic factors, lifestyle or cultural factors, environmental factors, and variability in hormone exposures. For example, a previous study found that the level of estradiol in black pregnant women is higher than that in whites, but the increase in testosterone levels was even more significant [34]. Furthermore, the ratio of estradiol to testosterone was significantly lower in blacks compared to whites. Therefore, differences in hormone levels between pregnant women in different races may be a source of differential exposure across racial groups, thereby influencing TC risk. In addition, a case-control study of diet and testicular carcinoma found that higher total fat consumption was borderline significantly associated with increased mixed germ cell tumor risk [35]. Similarly, heterogeneity in the dietary structure of different races may also be a contributing factor to the observed differences in TC incidence. In the Western diet structure, fat intake is relatively high, accounting for 39% (35 to 45%) of total calories. However, in the Eastern diet structure, fat only accounts for approximately 20% of total calories, which may help explain why the incidence of TC among whites is higher than that of other racial groups. Previous study on dietary structure have shown that black people’s dietary structure is lower than white people’s in terms of fat intake, and the sweets/fat dietary pattern were more likely to be male, White, with lower education and income [36].

Previous publications have also reported on the racial differences of TC patients with respect to survival outcomes [37–39]. Bridges et al conducted a 14-year review of 215 consecutive American patients with testicular cancer and calculated the actuarial 5-year survival rates at 88% in white patients and 71% in black patients [40]. Judd et al found that race was significantly associated with testicular cancer death, with non-Caucasian men being 1.69 times more likely to die of testicular cancer than Caucasians on univariate analysis. Historically, non-Caucasian race has been associated with poorer outcomes from testicular cancer [41]. In the current analysis, we observed that whites had the highest survival rates, while blacks had the worst survival rates; suggesting that white men had a survival advantage over other races. Also consistent with the previous reports, we found that the survival rates among HWs and APIs were quite similar to one another (92.2 and 91.8%, respectively). The observed differences in survival may be due to cultural attitudes regarding malignancy, and/or knowledge and perceptions around cancer screening. At the same time, access to health care and the dissemination of health information are also potentially subjective explanations for why the survival rates of whites are higher than that of blacks [42]. Previous study found that for testis tumor treated at the same institution, there was an increased delay of diagnosis in blacks compared with whites, and the incidence of this tumor in blacks does not appear to be increasing [43]. Another study also compared prognostic data between Asians and whites, finding that Asians had lower survival rates, possibly as a result of diagnostic and therapeutic differences [44].

In our study, we found the age at diagnosis was significantly different across racial groups. HWs had the youngest age at diagnosis (30.8 years) and NHWs had the oldest (36.9 years). Several explanations may account for the racial differences observed in the current study and in previous reports [19, 45, 46]. For example, race-specific perceptions of disease, differences in socioeconomic status, the availability of health knowledge, and differences in health care accessibility may lead to differences in disease detection and may influence treatment options. When diagnosed at a localized or regional stage, TC prognosis is usually good, with survival rates as high as 95%. As noted elsewhere, with continued improvements in TC treatment, the therapeutic response of early testicular cancer is extremely favorable [47]. However, in patients diagnosed with distant stage TC, the five-year survival rates drop dramatically, particular among blacks (72.5%). These findings further highlight the importance of early detection.

There are also a few publications that have studied tumor location of TC with respect to cancer incidence and prognosis. According to these studies, undescended testis is a risk factor for TC and is usually treated surgically, but whether the age at treatment has any influence on risk remains unclear [48]. In this study, we analyzed the connection between survival rates and tumor location. When stratified by tumor location, TC in the descended testis is much more common than that of undescended testis. NHWs have the highest survival rates (96.7%) for TC in descended testis whereas API have the best survival rates (95.8%) for undescended testis. The racial differences were significant for all tumor locations. The causes of these differences are not clear at present, and therefore require further study.

We also observed significant difference among race groups according to TC treatment strategy. For patients receiving chemotherapy alone or combined chemotherapy and radiation, the treatment efficacy among NHWs is obviously superior to that observed among APIs and blacks. For radiation alone and combined surgery and radiation, HWs had the best survival rates, while for chemotherapy alone, or chemotherapy in combination with surgery or radiotherapy, NHWs had the highest survival rates (90.7, 85.6, and 63.5%, respectively). In general, the treatment of testicular tumor is divided into surgical treatment, radiotherapy alone, chemotherapy alone, and combined treatment [49]. Once identified as a testicular tumor, radical testicular resection should be performed first, and further treatment should be decided upon according to the results of the subsequent pathological examination. The diverse treatment regimens may have important impacts on prognosis, while at the same time, racial differences in post-treatment survival may result from differences in health status and the presence of comorbidities. The landmark findings detailed in the Secretary’s Task Force Report of Black and Minority Health in 1985 [50] revealed significant differences in access to medical care by race and ethnicity within certain disease categories and by various types of health services. Adherence of blacks and APIs to post-operative review and follow-up regimens is not as high as that of whites, which requires further study, alongside other potential explanations for the observed racial differences in survival.

We used the SEER database for analysis since it is currently the longest-serving cancer registry in the United States. The data of SEER registry showed that White, Black, Asian and Hispanic cover 33.6, 34.7, 62.6, and 46.7% of the respective ethnic population [51]. The proportion of Asian is a little higher than other ethnic groups, but we don’t think it affected the results according to the huge sample size. However, there are some limitations inherent in this data. First, since data is initially collected at multiple sites by several individual registries, there is the potential for administrative errors in the recording of tumor classification and staging. However, since we do not expect these types of errors to be systematic or correlated with ethnicity, any impact on our findings is likely to be relatively modest. Second, while SEER represents a large, population-based dataset, the information provided may still be inadequate for analyzing some aspects of tumor biology. For example, the lack of data on genetic factors preclude the possibility of analyzing disease subsets with distinct genetic etiology, or the impact of gene-environment interactions on TC risk and prognosis. In addition, the information on ethnicity and geographical origin is relatively cursory, and relies on self-report. Moreover, treatment selection can be affected by many factors not measured in the dataset, including insurance status, treatment availability, and others. Also, the SEER data only contain information on patients from the United States, the proportion of various ethnic groups on SEER data is basically as same as that of the 2010 U.S. Census [50]. So we think the results will represent the TC epidemiology situation in the United States. But it is not clear whether the results of this study would hold true for other regions. In addition, although the sample size is at the same level as in related studies [38, 52], it would possible be over powered.

## Conclusions

In summary, analysis of SEER data suggests that significant racial differences exist among TC patients in the United States in terms of patient characteristics, incidence, and survival. However, the exact explanations for such differences remain to be elucidated. In spite of the study’s limitations, our results can be useful to TC epidemiologists, clinicians, policy makers, and other stakeholders interested in reducing the burden of TC morbidity and mortality.

## Supplementary information

**Additional file 1 **: F**igure S1.** Trend of incidence rate from 1973 to 2012, stratified by racial group. (NHW: non-Hispanic whites. HW: Hispanic whites. API: Asians and Pacific Islanders.)

**Additional file 2 **: **Figure S2.** Trend of 5-year relative survival rate from 1973 to 2007 with follow-up until 12/31/2012, stratified by racial group. (NHW: non-Hispanic whites. HW: Hispanic whites. API: Asians and Pacific Islanders.)

## Data Availability

The datasets generated and/or analysed during the current study are available in the Surveillance, Epidemiology, and End Results Program repository, https://seer.cancer.gov/data/.

## Abbreviations

TC: Testicular cancer
SEER: Surveillance, Epidemiology, and End Results
NHW: Non-Hispanic whites
HW: Hispanic whites
API: Asians and Pacific Islanders
GCTs: Germ cell tumors
EC: Embryonal carcinoma
CS: Chemotherapy plus surgery
RS: Radiotherapy plus surgery
CRS: Chemotherapy plus radiotherapy plus surgery
CR: Chemotherapy plus radiotherapy
