# Supplementary figures and images for: Racial differences in testicular cancer in the United States: descriptive epidemiology
Source: BMC Cancer. 2020 Apr 6;20:284. doi: 10.1186/s12885-020-06789-2 (PMC7137202; doi:10.1186/s12885-020-06789-2)

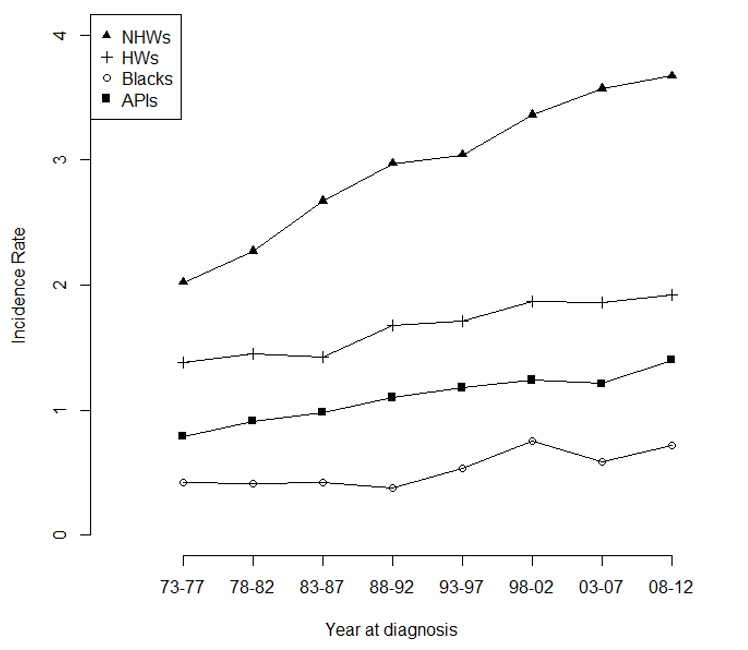

Supplement: Supplementary file 1 — Additional file 1 : Figure S1. Trend of incidence rate from 1973 to 2012, stratified by racial group. (NHW: non-Hispanic whites. HW: Hispanic whites. API: Asians and Pacific Islanders.) [file 12885_2020_6789_MOESM1_ESM.docx]

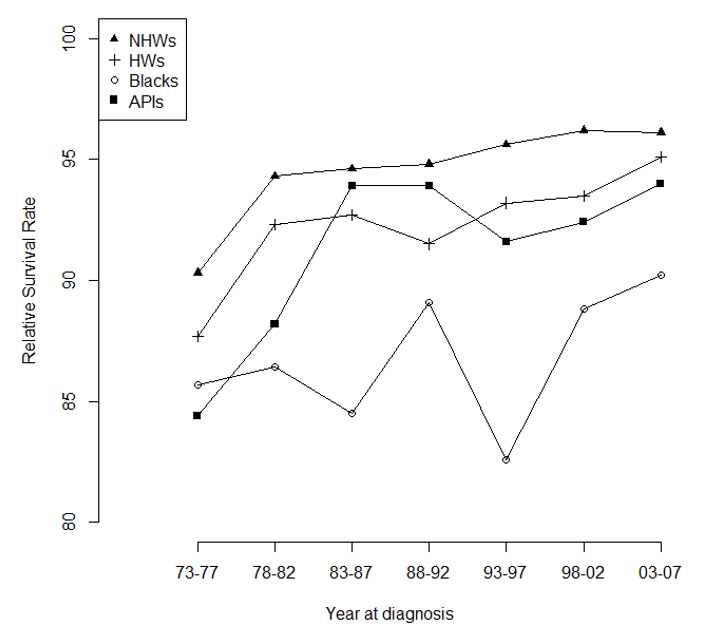

Supplement: Supplementary file 2 — Additional file 2 : Figure S2. Trend of 5-year relative survival rate from 1973 to 2007 with follow-up until 12/31/2012, stratified by racial group. (NHW: non-Hispanic whites. HW: Hispanic whites. API: Asians and Pacific Islanders.) [file 12885_2020_6789_MOESM2_ESM.docx]
